# Methodologies to Assess the Bioactivity of an Herbal Extract on Immunity, Health, Welfare and Production Performance in the Chicken: The Case of *Melissa officinalis* L. Extract

**DOI:** 10.3389/fvets.2021.759456

**Published:** 2021-10-22

**Authors:** Angélique Travel, Angélique Petit, Perrine Barat, Anne Collin, Camille Bourrier-Clairat, Marion Pertusa, Fabien Skiba, Sabine Crochet, Estelle Cailleau-Audouin, Pascal Chartrin, Vanaïque Guillory, Denis Bellenot, Rodrigo Guabiraba, Laurence A. Guilloteau

**Affiliations:** ^1^Institut technique des filières avicole, cunicole et piscicole, Nouzilly, France; ^2^Institut national de recherche pour l'agriculture, l'alimentation et l'environnement, Université de Tours, Biologie des oiseaux et aviculture, Nouzilly, France; ^3^Nutricia, Haut Mauco, France; ^4^nstitut national de recherche pour l'agriculture, l'alimentation et l'environnement, Université de Tours, Infectiologie et santé publique, Nouzilly, France; ^5^Institut technique interprofessionnel des plantes à parfum, médicinales et aromatiques, Chemillé-en-Anjou, France

**Keywords:** herbal extract, *Melissa officinalis*, poultry, innate immunity, welfare, health, performance, methodology

## Abstract

The potential of herbal extracts containing bioactive compounds to strengthen immunity could contribute to reducing antimicrobial use in poultry. This study aimed at developing a reliable and robust methodological pipeline to assess the ability of herbal extracts to strengthen chicken innate defenses, especially concerning inflammation and oxidative stress. This methodology was applied to *Melissa officinalis* L. (MEL) extract, recognized for its biological activities including antioxidant and anti-inflammatory properties. Different methods were used to (1). guarantee the quality of MEL extract and its capacity to stimulate the innate immune system; (2). evaluate the relevance of an *ex vivo* model to mimic inflammatory and oxidative stress challenges to replace LPS injection in chickens; (3). analyse the effects of feed supplemented with MEL extract on inflammation and oxidative stress induced *ex vivo*; (4). assess the effects of MEL extract on the redox balance, health, welfare and performance in broilers exposed to suboptimal starting conditions through a large-scale approach. The quality of MEL extract preparations, through phytochemical quantification of rosmarinic acid (RA), revealed varying concentrations of RA in the different MEL extracts. RA concentrations remained stable for at least 9 months and in feed three months after incorporating MEL extract. When incubated with chicken cell lines MEL extract showed potential metabolic activation and ability to stimulate immune functions but induced cytotoxicity at high concentrations. The original *ex vivo* model of inflammation developed on chicken blood cells enabled inflammation and oxidative stress biomarkers to be expressed and revealed antioxidative and anti-inflammatory properties of blood cells from chickens fed MEL extract. The experimental model of chicken suboptimal starting conditions validated beneficial effects of MEL extract on the redox balance and also evidenced improved performance during the growth phase, a tendency for fewer muscle defects but a higher severity of pododermatitis lesions without affecting other welfare indicators. This study grouped methods and tools that could be combined according to the plant extract, the needs of professionals working in poultry production systems and staff responsible for animal health, welfare and feeding.

## Introduction

In poultry production systems, broiler chicks are exposed to various stress factors from hatching to the first week of life. Stress during early life can induce persistent changes in physiology, behavior, immunity and consequently in overall chicken health (1–4). Chicks have outstanding robustness and resilience to environmental disturbances, i.e. the ability to maintain or regain a state of dynamic equilibrium after a period of imbalance. However, the genetic selection based on improving performance has negatively affected these capacities in fast-growing broilers.

Innate immunity is the most efficient protective response in early life. In response to biotic or abiotic stressors, the organism promptly produces substances such as cytokines/chemokines, lipid mediators, reactive oxygen and nitrogen species (ROS/RNS) which are mediators of inflammation and oxidative stress. Oxidative stress is the result of an imbalance between oxidative and antioxidative activities within the cells. It is a physiological process involved in the maintenance of cell integrity, with numerous functions in immunity and inflammation. Inflammation and oxidative stress are naturally regulated. However, they can become persistent and lead to chronic, low-grade inflammation and deleterious effects on cells, tissues and their functions (5). To balance the redox status, animals maintain a complex system of endogenous antioxidants, including enzymes (e.g. glutathione, catalase and superoxide dismutase, etc.), proteins and low molecular weight scavengers, such as uric acid. The endogenous antioxidant defense system is complemented by exogenous antioxidants present in the diet or in feed supplements (e.g. vitamin E, vitamin C, phenolics (polyphenols, flavonoids) and carotenoids).

One strategy to support the adequate functioning of the chick immune system in early life is to supplement their feed with plants containing bioactive compounds operating in the body defense systems. Growing concerns about the increase of antimicrobial resistance in farm animals led to changes in EU legislation governing the use of antibiotics as a growth factor in poultry feed, which resulted in their suppression in 2006 (Council Directive 96/22/EC; Axis 2 and measure 19 of the EcoAntibio 2017 plan). This decision led the livestock industry to search for new solutions to maintain poultry health. For decades, herbal extracts have been known for their antioxidant, anti-inflammatory and antimicrobial properties in humans and livestock (6, 7). Herbal extracts are already used as additives in poultry feed to improve performance and the quality of end products (8–11). However, the validation of herbal extracts as an added value for health in poultry production remains limited due to the lack of literature with accurate and fully documented methodologies to evaluate their biological effects.

The choice of herbal extracts from the scientific literature is also challenging due to the lack of details on the phytochemical characterization and experimental methods employed. Grids for evaluating scientific publications and experiments concerning herbal extract, and not essential oils, quality and biological activity for a given application have been developed (12). *Melissa officinalis* (MEL) belongs to the Lamiaceae family and is native to the eastern part of the Mediterranean basin and western Asia. It is currently used in different ethnomedical systems e.g. botany, phytochemistry, pharmacological activities, safety and clinical applications. Modern pharmacological reviews reported that MEL has several biological activities including antioxidant, hypoglycemic, hypolipidemic, antimicrobial, anticancer, antidepressant, anxiolytic, anti-inflammatory and spasmolytic properties (13–15). Phytochemical investigations on MEL have revealed the presence of bioactive substances including volatile compounds (e.g. geranial, neral, citronellal and geraniol), triterpenes (e.g. ursolic acid and oleanolic acid), and phenolic compounds (e.g. rosmarinic acid isomers, caffeic acid derivatives, luteolin and quercitrin) (14, 16–18). Nevertheless, only a few studies have assessed the capacity of MEL to improve the defense system of poultry and their production performance.

Therefore, this study aimed at developing a reliable and robust methodological pipeline and tools to assess the ability of herbal extracts, here applied to MEL extract, to strengthen the innate defenses in chickens. The main objectives were to (i) guarantee the quality of MEL extract and its capacity to stimulate the innate immune system, (ii) evaluate the relevance of an *ex vivo* model to mimic an inflammatory and oxidative stress challenge to replace the LPS injection model in chickens (iii) analyse the effects of poultry feed supplemented with MEL extract on inflammation and oxidative stress induced *ex vivo*, and (iv) appreciate the effects of MEL extract on the redox balance, health, welfare and performance in broilers exposed to suboptimal starting conditions through a large-scale approach.

## Materials and Methods

### MEL Extracts and Feed Preparations

Extracts from the dried leaves of MEL were purchased from local suppliers (Pharmanager Ingredients, Angers, France and EVEAR Extraction, Brissac Loire Aubance, France). MEL extracts were declared to contain 5% rosmarinic acid (RA), free of *Salmonella, Escherichia coli*, allergenic substances and compounds of animal origin. Residual pesticide levels were in accordance with the European regulation 396/2005 and its amendments.

### Chemical Characterization

The phytochemical characterization of MEL extracts was carried out at the ITEIPMAI (Chemillé en Anjou, France). RA content was measured in dry extracts of MEL leaves using a chromatographic method described in European Pharmacopeia (01/2010:2524), and adapted for fluorimetric detection and for feed mash and pellet. To this end 0.200 g of RA was extracted using ultrasonification in 50 mL of ethanol-H_2_O (1:1 v/v), for 10 min at room temperature. After filtration into a 100 mL volumetric flask and stabilization at 20°C, the solution was completed to 100 mL with the same solvent. To determine the RA content in the mash and pellets, 10 g were extracted by ultrasonification in 50 mL of ethanol-H_2_O (1:1 v/v), for 15 min at room temperature. After decantation, the supernatant (about 5–6 mL) was centrifuged for 10 min at 3,000 rpm. The supernatant was transferred to a vial for chromatography. The reference solution was prepared with 20 mg of RA dissolved by ultrasonification in 50 mL of ethanol-H_2_O (1:1 v/v) for 10 min at room temperature, completed with 100 mL and then diluted 1:5 (v/v) with ethanol-H_2_O. The detection of RA was performed using high-performance liquid chromatography with diode array detection (HPLC-DAD, Agilent 1,260 analyser) and HPLC combined with fluorescence detection (HPLC-FLUO, Shimadzu, LC-2040C 3D Nexera-i Plus with an RF-20Axs detector). For both methods, the column used was a C_18_ column (NUCLEOSIL, 5 μm, 250 mm ^*^ 4.6 mm, 100 Angstrom; Macherey Nagel, France), the mobile phase a gradient of two solvents: solvent A was a mixture of concentrated phosphoric acid (purity 85% m/m), acetonitrile and distillated water, in the following proportions 10/190/800 (v/v/v); solvent B was a mixture of concentrated phosphoric acid (purity 85% m/m), methanol and acetonitrile, in the following proportions 10/400/590 (v/v/v). The gradient was as following: from 0 to 20 min, from 100% A to 55% A; from 20 to 25 min, from 55% A to 0% A; from 25 to 30 min, from 0% A to 100% A. The flow rate was 1.2 mL/min at 25°C and the injection volume, 20 μL. For the HPLC-DAD method, the limit of detection was estimated to be 0.02 μg/mL in the reference solution. The dry matter (DM) content was measured (European Pharmacopeia, 07/2019:20232) and the results were expressed as RA % DM.

The MEL extract was also analyzed using gas chromatography (GC) - mass spectrometry (MS) after a prior derivatization as described before (19). A nuclear magnetic resonance (NMR) spectroscopy study was performed in parallel for a semi-quantitative evaluation of some compounds. To assess the stability of MEL extracts over time, the content of RA in MEL extract samples was determined and repeated 4 and 9 months after the first analysis.

### Feed Preparation

Feed was manufactured by TECALIMAN (Nantes, France) and Sud-Ouest Aliment factory (Haut Mauco, France). The pellet preparation was performed at 70°C. The composition of the basal feed for each breeding period, is described in Tables 1, **3**. Based on references (20, 21), MEL extract was supplemented at 1% (10 g/kg) into the mash which was then granulated. To evaluate the impact of the granulation process, the supplement rate of RA and the recovery rate in mash and pellets were measured using HPLC-DAD and HPLC-FLUO methods.

**Table 1:** Composition of the basal feed of chickens during starter (D1–10) and grower-finisher (D11–35) periods (Design 1).

**Ingredients (%)**	**Starter**	**Grower-Finisher**
Wheat	29.9	30
Corn	26.9	32.5
Soyabean grain	2.5	
Soyabean meal	32.3	27.8
Soyabean oil	2	2
Palm oil		1.9
Sodium bicarbonate	0.1	0.1
Phosphorus bicarbonate	1.8	1.5
Calcium carbonate	0.6	0.8
Sodium chloride	0.3	0.3
DL-Methionine	1.9	1.6
L-Lysine	0.7	0.7
Threonine	0.6	0.5
Premix^*^	0.4	0.4
**Calculated nutrient content (g/kg DM)**		
ME^**^ (kcal/Kg)	2,950	3,100
Crude Fat	4.3	5.8
Crude Protein	21.7	19
Ash	5.8	5.6
Calcium	1	1
Phosphorus available	0.5	0.4
Vitamin E (UI)	80	80

* *Premix, provided per kg of diet: 2,500,000 UI vitamin A; 1,000,000 UI cholecalciferol; 20,000 UI DL-α-tocopherol; 1,000 mg menadione; 1,000 mg thiamine; 4 mg propyl gallate*.

** *ME calculated from a correspondence table internal to the feed manufacturer*.

### Feeding Behavior

To evaluate the impact of MEL extract supplementation on the feeding behavior of chicks and their body weight, a preliminary assay was carried out for one week after hatching. The feed consumption and body weight were compared between chicks fed with a basal diet supplemented with 2% of MEL extract and those fed with a basal diet.

### Cell Culture With MEL Extract and Bioassays

To assess the potential cytotoxicity and immunostimulant properties of the MEL extract, chicken macrophage and hepatocyte cell lines were used as *in vitro* models due to their roles in chicken immunity and metabolism.

### Cell Culture

The HD11 macrophage cell line, an avian myelocytomatosis virus (MC29)-transformed chicken macrophage-like cell line (22), was cultured in RPMI1640 medium (Gibco, UK) supplemented with 10% heat-inactivated fetal calf serum (FCS, Gibco, UK), 10 mM HEPES, 2 mM glutamine, 100 U/mL penicillin and 100 μg/mL streptomycin and grown routinely in a 75 cm^2^ flask at 41°C under 5% CO_2_. The LMH avian hepatocyte cell line (23) was cultured in DMEM/Ham's F12 (1:1) medium (Gibco, UK) supplemented with 10% FCS, 10 mM HEPES, 2 mM glutamine, 100 U/mL penicillin and 100 μg/mL streptomycin and grown routinely in a gelatin-coated 75 cm^2^ flask at 41°C under 5% CO_2_.

Prior to any *in vitro* experiment, MEL extract was diluted in cell culture medium (1 mg/L) and tested for sterility in liquid brain heart infusion (BHI) growth medium, a nutrient-rich medium used to culture a wide variety of fastidious organisms. No bacterial growth was observed after 5 days of incubation (data not shown).

### Cellular Metabolic Activity

To evaluate the cytotoxic effects of different concentrations of the MEL extract (100 μg/mL-10 ng/mL), HD11 cells and LMH cells were seeded at 5 x 10^4^ cells/well in a 96-well culture plate and exposed to culture medium (control group) or MEL extract. At 6, 24 and 48 h after incubation, cellular metabolic activity was determined using the colorimetric methylthiazoletetrazolium bromide (MTT) assay (Sigma-Aldrich, UK). Briefly, MTT was added to a final concentration of 5 μg/mL per well and cells were incubated for 2 h at 40°C under 5% CO_2_. After complete solubilisation of the dye using DMSO, plates were read at 550 nm in a Multiskan Ascent plate reader (Thermo Fisher Scientific, USA). Absorbance values for the control group were set to 100% of metabolic activity.

### Nitric Oxide Production

Supernatants from the HD11 cell line exposed to different concentrations of MEL extract were harvested at 24 and 48h and tested with a nitrite (NaNO_2_) assay, as an index of nitric oxide (NO) production. Nitrite concentration was determined by spectrophotometry in cell culture supernatants using a standard Griess assay following the manufacturer's instructions (Promega, UK). The absorbance was read at 550 nm in a Multiskan Ascent plate reader (Thermo Fisher Scientific, USA). The nitrite concentration was calculated using a standard curve of sodium nitrite.

### NFκB Activity

Activation of NFκB-related signaling pathways by the MEL extract was assessed in a HD11-NFκB luciferase reporter cell line (24). Cells were routinely cultured in DMEM F-12 (1:1) medium (Gibco, UK), supplemented with 10% heat-inactivated FCS, 15 mM HEPES, 2 mM L-glutamine, 100 U/mL penicillin, 100 μg/mL streptomycin and 5 μg/mL puromycin (Sigma-Aldrich, UK), and incubated as described above. HD11-NFκB reporter cells were seeded at 2.5 x 10^5^ cells/well in 24-well plates and incubated at 41°C under 5% CO_2_ overnight. The next day, HD11-NFκB cells were incubated for 6 or 24 h with different concentrations of MEL extract or LPS at 10 ng/mL (from *E. coli* O:55 B:5, Sigma-Aldrich, France) as a positive control. Luciferase activity was measured using the luciferase assay reagent (Promega, USA) and a GloMax-Multi Detection System (Promega, USA). Data are expressed as NFκB activity (fold increase relative to the control group).

### Chickens and Experimental Models of Inflammation (Design 1)

The project (APAFIS#17516-201811132143782 v2) was evaluated and approved by the local Ethics Committee N°019 (Comité d'Ethique en Expérimentation Animale Val de Loire, Tours, France).

#### Animal Model

A first experiment was done with 12 male Ross PM3 broiler chickens to choose the most suitable method (*ex vivo* or *in vivo*) to test the effects of MEL in regulating the inflammatory response induced by *E. Coli* LPS (055:B5, Sigma-Aldrich, France). LPS was diluted in sterile endotoxin-free DPBS 1X (Gibco, UK) to a final concentration of 5 mg/mL.

In the *ex vivo* procedure, blood samples from occipital sinuses of 29-day-old chickens were collected in vacutainer tubes containing ethylene diamine tetra acetic acid (EDTA). Each blood sample was diluted (1/2) in complete culture medium containing DMEM (Gibco, UK), 2 mM L-glutamine, 10% FCS (Thermo Scientific, France) and 1% antibiotic and antifungal solution (A5955, Sigma-Aldrich, France) composed of penicillin (10 U/mL), streptomycin (10 μg/mL) and amphotericin B (25 pg/mL). After distributing the blood in wells containing complete culture medium alone (control group) or with LPS (10 μg/mL), the plate was incubated for 6 h at 41°C and 5% CO_2_. The supernatants were collected and stored at −80°C. Then 100 μL of blood cells were immediately diluted in 1 mL of TRIzol (Invitrogen™ LS15596018, Thermo Fisher Scientific, France) and vigorously shaken for 5 min on ice and stored at −80°C.

For the *in vivo* procedure, 31-day-old chickens each received a subcutaneous injection of LPS (at the level of the wishbone), at a concentration of 100 μg/kg body weight (25). Blood samples were taken before LPS injection (T0) and after 6 h (T6). The blood was centrifuged for 10 min at 2,000 g and 4°C. Plasma and cell pellets were collected and frozen at −80°C as described above.

A second experiment was done to evaluate the impact of MEL extract supplementation *in vivo* on regulating the inflammatory response and oxidative stress using the *ex vivo* procedure. At the Experimental Poultry Facility (PEAT, INRAE, Nouzilly, France, https://doi.org/10.15454/1.5572326250887292E12), 24 male Ross PM3 one-day-old chicks hatched at Boyer (La Boissière en Gatine, France) were placed into two floor pens (3m × 1m) of 12 chicks each (i.e. a density of 4 animals/m^2^). Throughout the rearing period, the birds had access to water and feed *ad libitum*. Starter (D1–D10), Growth/Finisher (D11–D35) diets were supplemented with MEL in a proportion equivalent to 1% of the basal diet for only one of the two pens. The birds were weighed at 1, 6, 12, 19, 27 and 35 days of age. Blood samples were collected in vacutainer tubes containing EDTA from the occipital sinus at 14 and 30 days of age and assessed for blood cell reactivity to LPS using the *ex vivo* procedure as described above. Blood samples were also collected at 34 days of age and plasma was stored at −80°C for later physiological parameter analyses.

#### Physiological Parameters

Antioxidant and oxidative status as well as metabolic and inflammatory parameters were measured at 34 days after hatching in the blood of chickens receiving or not MEL extract. Commercial kits (Thermo Fisher Diagnostics SAS, France) were used to determine plasma glucose (mg/L), uric acid (mg/L) and triglyceride (mg/L) concentrations. Total plasma antioxidant activity was determined through total antioxidant status (TAS) measurement (mmol/L) (Randox Laboratories, UK). The enzyme activity involved in antioxidant defense such as superoxide dismutase (SOD) and glutathione peroxidase (GPx) was measured with commercial kits (Sigma-Aldrich, Lyon, France and Randox Laboratories, London, United Kingdom respectively). The haptoglobin-like activity (mg/mL), which increases in response to acute infection or inflammation, was measured using a commercial kit (Tridelta Development Limited, Maynooth, Ireland). Protocols listed above were used in accordance with the supplier instructions and adapted to the Thermo Scientific Arena 20XT photometric analyzer (Thermo Fisher Scientific, Courtaboeuf, France). The concentration of total glutathione (oxidized and reduced forms, μM) was measured in a spectrophotometer (TECAN infinite® 200, Männedorf, Switzerland) using a commercial kit (Ref 703018, Cayman Chemical Company, Michigan, United States). Lipid peroxidation was determined in the plasma using spectrophotometric measurement (UV mc^2^ Safas, Monaco) of thiobarbituric acid reacting substances (TBARS) (26).

#### RNA Extraction and Gene Expression Analysis

The blood cell samples stored at −80°C were used for total RNA extraction according to Désert et al. (27) to analyse the expression of genes involved in inflammation and oxidative stress (IL-1b, IL-6, IL-8 and iNOS) in response to LPS. RNA concentration and purity were measured with a NanoDrop UV-Vis spectrophotometer (NanoDrop™ 2000, Thermo Fisher Scientific, France) at 260/280 nm absorbance. A DNase treatment (Invitrogen™ LSAM1906, Thermo Fisher Scientific, France) was performed in order to avoid DNA contamination. An aliquot of 2μg of total RNA was reverse-transcribed in cDNA with Superscript II (200 U, Invitrogen, THERMO FISHER DIAGNOSTICS) and random primers.

Real-time qPCR was carried out in a CFX-Connect Real Time System (Biorad, Marnes-la-Coquette, France). Primer sequences are presented in Table 2. Real-time qPCR analyses were conducted using 7.5 μL iQ™ SYBR®Green Supermix (170-8884, Bio-Rad, Marnes-la-Coquette, France), 0.75 μL of each primer (10 μM), 2 μL template cDNA and 4 μL RNase-free water. Target genes were amplified using the following thermocycler programme: 95°C for 3 min, 39 PCR cycles at 95°C for 15 s and at an optimized melting temperature (Tm) for 40 s (Table 2), and final melting curve. The efficiency of amplification was established for each primer pair by utilizing the serial dilutions of cDNA and each sample was run in duplicate. Hypoxanthine-guanine phosphoribosyltransferase (HPRT) and ribosomal protein S8 (RPS8) were selected as reference genes. The quantification of PCR reactions for each primer pair was carried out by comparing the target gene with the reference genes for which a normalization factor had been calculated using the GeNorm software (Microsoft Excel GeNorm algorithm, version 3.5, 2002) to establish the relative gene expression. The method below was used to compute the gene expression of the target gene according to the method described by Pfaffl (28) and Vandesompele et al. (29):


$$\begin{array}{l} \text{Ratio}={\left(E_{\text{target}}\right)}^{\text{Δ}CT\;target\;\left(Medium-LPS\right)}/\\ {\left(E_{\text{reference}}\right)}^{\text{Δ}CT\;reference\;\left(Medium-LPS\right)} \end{array}$$

^*^E =Expression^*^Ct = Cycle Threshold.

**Table 2:** Primer sequences used in real-time PCR analyses.

**Target gene**	**Sequence (F: forward, R: reverse)**	**GenBank access**	**PCR fragment lenght (pb)**	**Tm (^**°**^C)**
IL-1β	F: 5′-AGGCTCAACATTGCGCTGTA-3′	XM_015297469.1	98	64
	R: 5′-CTTGTAGCCCTTGATGCCCA-3′			
IL-6	F: 5′-GCTTCGACGAGGAGAAATGC-3′	XM_015281283.2	139	62
	R: 5′-GCCAGGTGCTTTGTGCTGTA-3′			
IL-8(L2)	F: 5′-CTGCGGTGCCAGTGCATTAG-3′	NM_205498.1	139	62
	R: 5′-AGCACACCTCTCTTCCATCC-3′			
iNOS	F: 5′-CCACCAGGAGATGTTGAACTATGTC-3′	NM_204961.1	76	62
	R: 5′-CCAGATGTGTGTTTTCCATGCA-3′			
HPRT	F: 5′-TGGTGGGGATGACCTCTCAA-3′	NM_204848.1	177	65
	R: 5′-GGCCGATATCCCACACTTCG-3′			
RPS8	F: 5′-TGAGCGGAAGAAGAATGCCA-3′	NM_001252126.1	119	62
	R: 5′-ACACATAGCCATCAGCTCGG-3′			

*IL-1β, interleukin 1β; IL-6, interleukin 6; IL-8(L2), interleukin 8-like 2; iNOS, inducible nitric oxide synthase; HPRT, hypoxanthine-guanine phosphoribosyltransferase; RPS8, ribosomal protein S8*.

The results were presented as a fold change between LPS and medium of T6 and T0.

### The Effect of MEL in Chicken Reared Under Suboptimal Conditions (Design 2)

#### Experimental Design and Bird Management

The study was carried out in the Experimental Poultry Facility (NUTRICIA, Benquet, France) and was approved by the Animal Experimentation Ethics Committee N°073, Aquitaine Poissons Oiseaux (number APAFIS#20264-2019041117575067 v4). The study involved 1,440 1-day-old Ross PM3 male chicks obtained from a local hatchery (Socavic, 40500 Audignon, France). They were vaccinated against infectious bronchitis virus (BIORAL® H120 NEO, BOEHRINGER INGELHEIM, France) according to the supplier recommendations. Hatching eggs and chicks were placed under sub-optimal conditions, close to those that may be observed in commercial settings: long storage of eggs before incubation (18 days), no access to feed or water for 24h post-hatching at 18°C and high breeding density (39 kg/m^2^).

To assess the effects of MEL extract in the feed as an alternative to limit the deleterious effects of sub-optimal rearing conditions, two different dietary treatments with nine replicates each (40 chicks/replicate) were tested. Chicks were randomly assigned to 18 floor pens (1 m x 3 m). Pen bedding consisted of 10 cm-deep chopped straw. From the first day until the end of the experiment (31 days of age), chicks received a commercial diet (Control), which was formulated according to broiler nutritional requirements, or a commercial diet supplemented with a 1% MEL extract (10 g/kg feed). Vitamin E concentration in both diets was suboptimal (20 UI in the starting/growing period and 17 UI in the finishing period). The diets were manufactured at the Sud-Ouest Aliment factory (40280 Haut Mauco, France) and measurements of MEL concentration, durability, pellet hardness and crumb size were performed on all preparations. Ingredients of the experimental diets are listed in Table 3. Feed and water were provided *ad libitum* throughout the experiment and birds were maintained under a 18 L:6D photoperiod (30 lux). Temperature was monitored starting at 32°C on D1, gradually decreasing to 20°C at D20 until D31. Individual body weight (BW) and floor pen feed intake (FI) were recorded at D1, D11, D21 and D31 to calculate individual daily weight gain (DWG) and floor pen feed conversion ratio (FCR). Chicken mortality was monitored daily.

**Table 3:** Composition of the basal feed of chickens during the suboptimal experiment: starter (D1-11), grower (D12-21) and finisher (D22-31) periods (Design 2).

**Ingredients (%)**	**Starter**		**Grower**		**Finisher**	
	**Control**	**MEL 1%**	**Control**	**MEL 1%**	**Control**	**MEL 1%**
Wheat	34.9	35	35	35	35	35
Corn	26.9	25	28.4	27.9	33.7	31.8
Corn draff	3	3	4.5	4.5	6	6
Sunflower oil	1.2	1.9	1.9	2.0	1.6	2.2
Sunflower meal	0	0	1.2	0	0	0
Soyabean meal	29.7	29.9	20.9	23.1	20.9	21.2
Rapeseed meal (deoiled)	0	0	5	3.3	0	0
Salt	0.1	0.1	0.2	0.2	0.2	0.2
Calcium carbonate	1.3	1.3	0.9	0.9	0.9	0.9
Dicalcium phosphate	1.4	1.4	0.9	0.9	0.5	0.5
Sodium sulfate	0.4	0.4	0.2	0.2	0.1	0.1
DL-Methionine	0.3	0.3	0.2	0.2	0.2	0.2
L-lysine	0.3	0.3	0.4	0.3	0.3	0.3
Threonine	0.1	0.1	0.08	0.08	0.07	0.07
Choline Chloride 75	0.1	0.1	0.1	0.1	0.08	0.08
Anticoccidial	0.06	0.06	0.06	0.06	0.05	0.05
Xylanase	0.015	0.015	0.015	0.015	0.014	0.014
Phytase	0.015	0.015	0.015	0.015	0.015	0.015
Red pigment					0.015	0.015
Vitamin and mineral premix^*^	0.2	0.2	0.2	0.2	0.2	0.2
**Calculated nutrient content (g/kg DM)**						
ME^**^ (kcal/kg)	2,903	2,904	2,939	2,936	3,019	3,018
Crude Fat	3.4	3.9	4.3	4.3	4.2	4.8
Crude Protein	21.3	21.3	19.8	19.8	18.5	18.5
Ash	6	6.1	5.1	5.1	4.4	4.5
Calcium	1	1	0.7	0.7	0.6	0.6
Phosphorus available	0.6	0.6	0.6	0.5	0.5	0.4
Vitamin E (UI)	20	20	20	20	17	17

* *Premix, provided per kg of diet. Starter- Grower premix: 5,000,000 UI vitamin A; 2,000,000 UI cholecalciferol; 10,000 UI DL-α-tocopherol; 1,000 mg menadione; 800 mg thiamine; 6,900 mg Cu; 15,000 mg Fe; 35,000 mg Mn; 30,000 mg Zn; 750 mg K; 150 mg Se. Finisher premix: 4,000,000 UI vitamin A; 1,250,000 UI cholecalciferol; 8,700 UI DL-α-tocopherol; 800 mg menadione; 500 mg thiamine; 6,000 mg Cu; 15,000 mg Fe; 30,000 mg Mn; 27,500 mg Zn; 600 mg K; 150 mg Se*.

** *ME calculated from a correspondence table internal to the feed manufacturer*.

#### Chick Quality

At day 1 post-hatching, 40 chicks were randomly weighed and selected for quality measurement (30). Briefly, the Tona score of various parameters such as activity (/6), down and appearance (/10), retracted yolk (/12), eyes (/16), legs (/16), aspect of the navel area (/12), remaining membrane (/12) and remaining yolk (/16) were assessed. These characteristics were scored and added to calculate a total scale on a maximum of 100.

#### Welfare and Health Assessment

Poultry welfare and health were assessed with the EBENE® method indicators (31) based on the four principles of animal welfare assessment (good feeding, good housing, good health, appropriate behavior) and validated by several poultry professionals (selection, feed industry, farmers' organizations, veterinarians, farmers). Indicators were measured three times during the trial at D15, D23 and D28 except for footpad dermatitis, cloacal cleanness and respiratory problems that were observed three times at D11, D21 and D31. Two trained people each assessed one half of the floor pens by noting the following indicators: litter quality and number of dead birds since their arrival (and reasons). Then, each assessor placed in front of a pen counted the proportion of birds lying down without any activity and, for two min per floor pen, the number of broilers performing the following behaviors: foraging, leg/wing stretching or wing flapping, aggressive pecking and social interactions. The proportion of birds lying down without any activity was again marked. The assessor entered the floor pen and marked indicators related to broiler health: injury, immobile, lame or other abnormality. The footpad dermatitis assessment was performed according to the grid defined by Michel et al. (32). Cloacal cleanness (salt/clean) and respiratory problems (absence/presence) were performed at the same time. Each assessor observed 90 birds per treatment (10 birds in each floor pen).

#### Meat Quality Parameters

At the end of the experiment, broilers were sacrificed and the carcasses were prechilled and chilled before deboning. After deboning, breast filets were obtained. One-hundred breast filets per group were randomly selected and scored for meat defects such as white striping, wooden breast and ≪ spaghetti ≫ muscles (33). Meat defects were considered as indicator of performance and also as an indicator related to health.

#### Blood Samples and Physiological Parameters

Blood samples (three mL) were taken from the occipital sinus at D30 (18 chickens/group). Blood for measurements of physiological parameters was taken from the occipital sinus in heparinised tubes and centrifuged at 4°C for 10 min, 4,000 rpm. Plasma was collected and stored in microtubes at −80°C. Different markers of oxidative and inflammatory status were analyzed on plasma as described above. At D30, a few drops of blood were also taken from 16 chickens/group and smeared on two glass slides for analysis of their leukocyte formula. The smears were stained with Wright stain for 15 min (Selarl Veterinaires ABIOPOLE, Arzacq, France). On each slide, 100 including heterophils (H), lymphocytes (L), monocytes (M), basophils (B) and eosinophils (E) were counted and the H/L ratio was calculated by dividing the number of heterophils by that of lymphocytes (34).

### Statistical Analysis

Analyses were carried out using StatView Software program (version 5.0, SAS Institute, 1992–1998, Cary, NC) and XLSTAT software (version 2020.3.1, Addinsoft, Paris, France). Data of biochemical parameters on blood and cell lines, gene expression, H/L ratio and performance were analyzed by ANOVA after having checked the normality of residual distribution and the homogeneity of variances (Fisher test). When the residuals were not normally distributed and variances were not homogenous between groups, data were analyzed with non-parametric tests: a Kruskal-Wallis test followed by a Mann-Whitney test for two-by-two comparisons. For the frequency of muscle defects and pododermatitis scores, a Chi-square test was performed. Differences were considered to be significant when *p-*values were below 0.05, to be a tendency when *p-*values were between 0.05 and 0.1 and not significant (NS) when *p-*values were above 0.1. The values are presented as mean ± standard error.

## Results

### Chemical Characterization of MEL Extracts

GC-MS profiles after derivatization identified metabolites belonging to various chemical classes such as organic acids (malic, tartric, caffeic, rosmarinic and catechollactic acids) and most predominantly sugars and maltodextrin (data not shown). Measurements of RA by the standardized method revealed variable concentrations in the different MEL extracts used, ranging from 1.3 to 2.3% DM (Tables 4, 5). This RA proportion remained stable in the MEL extract for at least 9 months. After feed preparation supplemented with MEL extract, RA was still detectable in the same proportions in mash and pellets processed at 70 and 85°C. We selected 70°C as the temperature to prepare feed supplemented with MEL extract in the future experiments. RA content was also detected in other feed supplemented with MEL extract and used in both experiments 1 and 2 although at a lower proportion in experiment 2. The RA concentration in feed was still stable 3 months later after the MEL extract supplementation (Table 5).

**Table 4:** Proportion of RA in MEL extract and during the process of supplemented feed production.

**Preparation**	**RA**	
	**% DM**	**ppm**
MEL extract + T0	1.44	14400
MEL extract T + 4mo	1.37	13700
MEL extract T + 9mo	1.38	13800
Mash	0.015	149
Pellets 70°C	0.010	101
Pellets 85°C	0.009	92

*Quantification of RA was performed by HPLC-DAD (high-performance liquid chromatography with diode array detection). DM, dry matter*.

**Table 5:** Proportion of RA in MEL extracts and in supplemented feed.

**Preparation**	**Design 1**		**Design 2**	
	**HPLC-DAD**	**HPLC-FLUO**	**HPLC-DAD**	**HPLC-FLUO**
MEL extract	2.28^*^	2.22	1.32	1.34
Starter (mash)	0			
Starter (pellets)	0			
Starter + MEL (mash)	0.018	0.019		
Starter + MEL (pellets)	0.017	0.017	0.0081	0.0096
Grower (mash)	0			
Grower + MEL (mash)	0.017	0.020		
Grower + MEL (pellets)	ND	ND	0.0090	0.0102
Finisher + MEL (pellets)			0.0004	0.0015
MEL extract T + 3mo			1.23	1.27
Starter (pellets) + MEL, T + 3mo			0.0069	0.0077
Grower (pellets) + MEL, T + 3mo			0.0093	0.0101
Finisher (pellets) + MEL, T + 3mo			0.0002	0.0010

*Quantification of RA was performed by HPLC-DAD (high-performance liquid chromatography with diode array detection) and HPLC-FLUO (HPLC combined with fluorescence detection)*.

* *RA is expressed as % of dry matter (DM)*.

### Assessment of Metabolic Activity and Immunostimulant Properties of MEL Extract on Chicken Cell Lines

**Figure 1:**
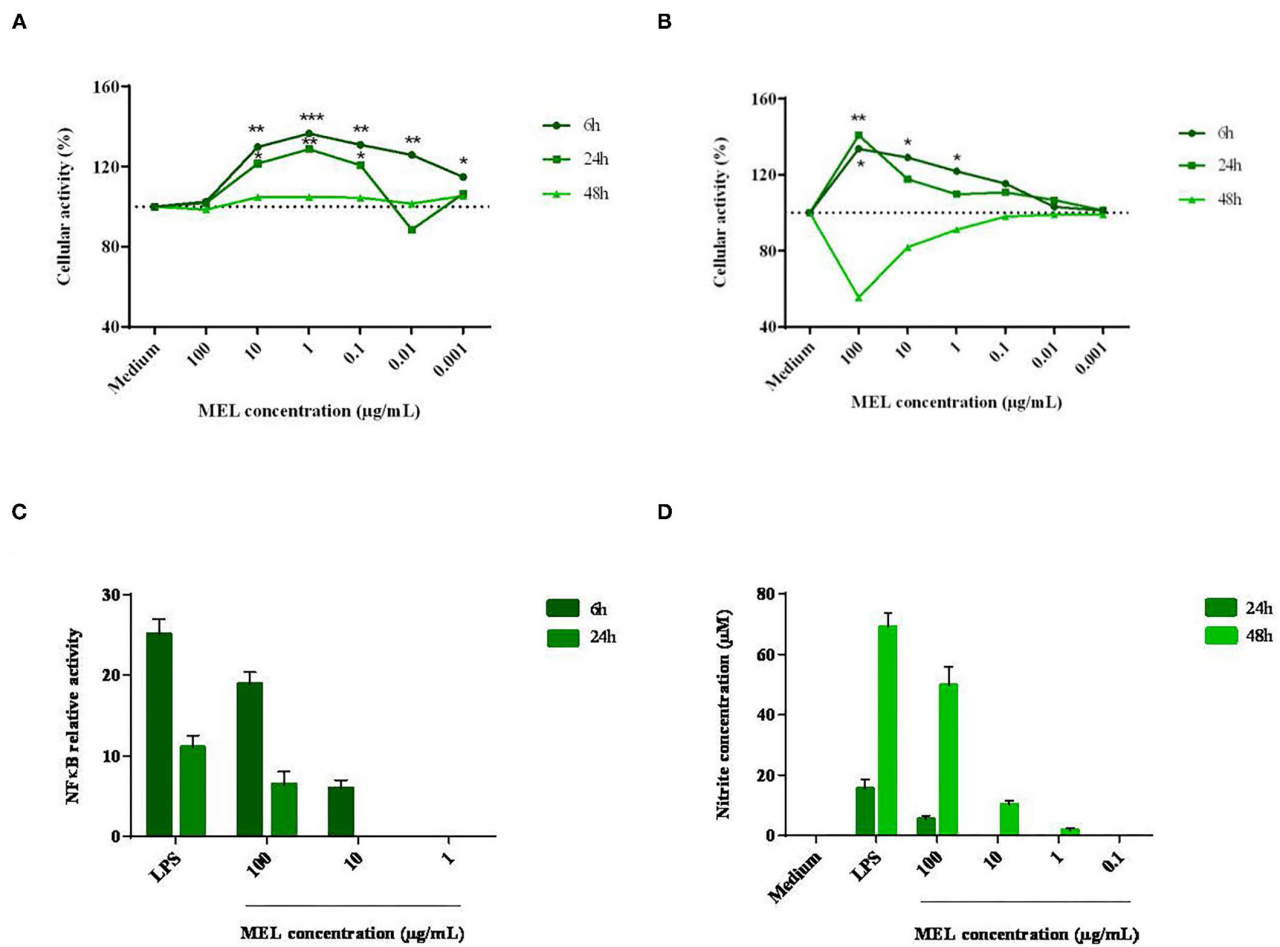
Effects of different concentrations of MEL extract on the activity of hepatocytes **(A)** and macrophages **(B)**, the relative activity of NFκB **(C)** and the nitrite concentration **(D)** after 6, 24 and 48 h of incubation. These parameters were measured using a MTT assay, standard Griess assay and luciferase test, respectively, in comparison with the growing medium containing or not an essential component of the wall of Gram-negative bacteria: the LPS. The data shown are means ± SEM (*n* = 3). Data were analyzed by ANOVA or with non-parametric Mann-Whitney test.

Cell viability and metabolic activity were assessed in two chicken cell lines representative of hepatic functions (LMH hepatocytes) and innate immunity (HD11 macrophages). From a concentration in the order of 10^−2^ mg/mL, MEL induced a significant increase in the metabolic activity of LMH cells, which could reach 40% after 6 h of incubation (Figure 1A). At 24 and 48 h, a loss of metabolic activity considered to be non-cytotoxic was observed. For HD11 macrophages, a high concentration of MEL (10^−1^ mg/mL) led to an increase in cell metabolism as early as 6 hours (Figure 1B). At lower concentrations, this effect was reduced. Similar kinetics was observed after 24 h of incubation. At 48 h of incubation, the metabolic activity decreased sharply (56%), suggesting a potential cytotoxic effect with a high concentration of MEL (> 50% loss in cell metabolism, ISO 10993-5:2009 - Biological evaluation of medical devices).

A potential immunomodulatory effect was then assessed in HD11 macrophages through the activation of the pro-inflammatory signaling pathway NFκB and the production of NO, a pro-oxidant and antimicrobial molecule. As seen in to Figure 1C, the activation of the transcription factor NFκB was 19-fold higher with MEL (10^−1^ mg/mL) than for the negative control (medium alone). LPS (positive control) induced a 25-fold increase compared to the negative control. Then, NO production was measured from 24 h of incubation (Figure 1D). At the highest concentration (10^−1^ mg/mL), MEL induced a 10-fold increase in NO production between 24 and 48 h of incubation (5 and 50 μM respectively). NO production was also observed at 48 h with lower concentrations (10^−2^ and 10^−3^ mg/mL). In comparison, LPS induced NO production in the order of 16 μM at 24 h. This response was multiplied by four at 48 hours (69 μM).

### Development of an *ex vivo* Model of LPS Challenge Using Chicken Blood Cells

**Figure 2:**
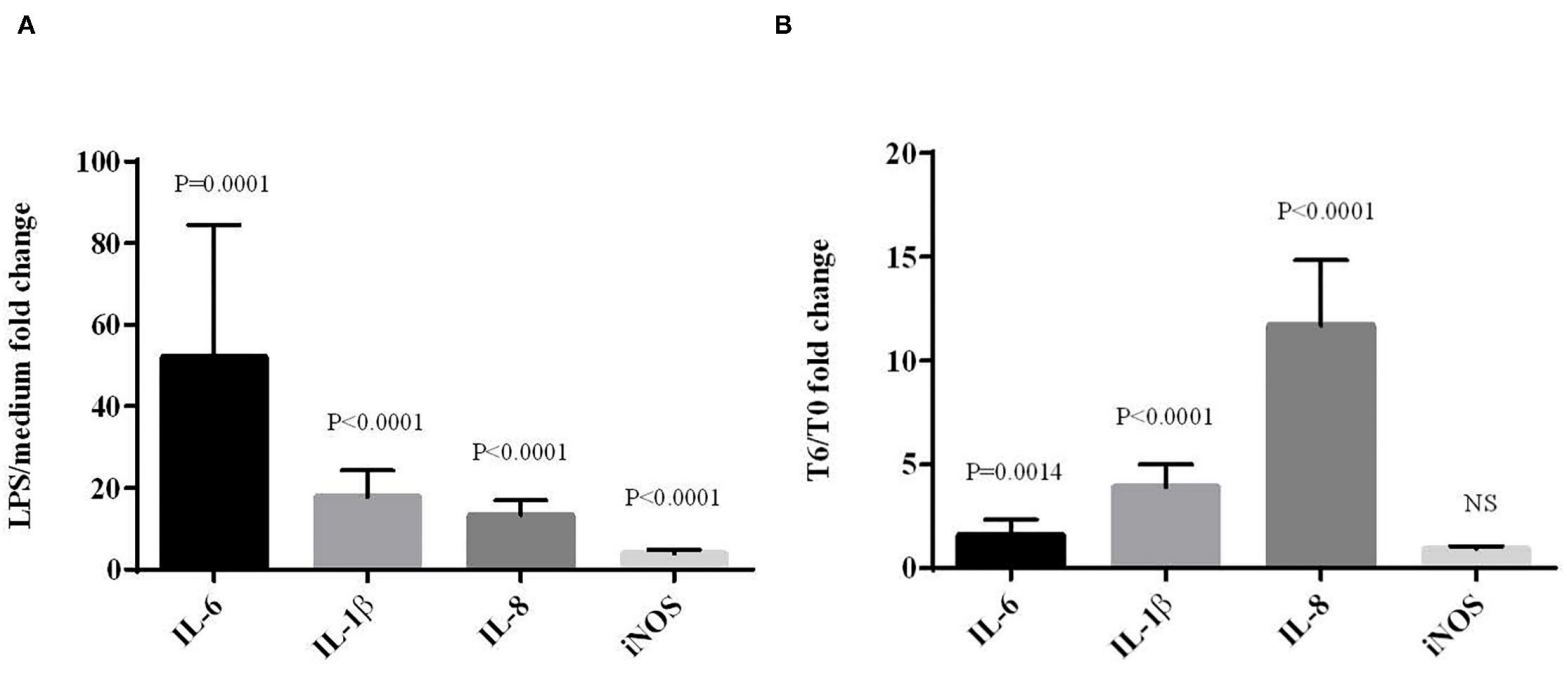
Effects of LPS on the expression of cytokine and iNOS mRNAs in chicken blood cells maintened for 6 h in culture (*ex vivo*) with LPS (10 μg/mL) **(A)** or from blood sampled 6 hours after subcutaneous LPS injection (100 μg/kg) (*in vivo*) **(B)**. mRNA expression was normalized to GAPDH, HPRT and RPS8 reference genes. Data represent mean ± SEM (*n* = 12). Data were analyzed by ANOVA or with non-parametric Mann-Whitney test. NS = *P* > 0.01.

Next, the LPS effects were evaluated on the induction of inflammation by analyzing the expression of genes coding IL-1β, IL-6, IL-8 and iNOS by RT-qPCR. For the *ex vivo* model (Figure 2A), mRNA levels of pro-inflammatory cytokines IL-1β, IL-6, IL-8 and iNOS were all upregulated in LPS-stimulated cells. The expression of the cytokine IL-6 was higher than the expression of IL-1β (*P* = 0.0166) and IL-8 (*P* = 0.0041). For the *in vivo* model (Figure 2B), the subcutaneous injection of LPS significantly increased the levels of IL-1β, IL-6 and IL-8 mRNA, but not that of iNOS between T0 and T6. As for the fold change, the expression of cytokine IL-8 was higher than the expressions of IL-1β (*P* = 0.0002) and IL-6 (*P* = 0.0002). The expression of IL-1β was significantly higher than that of IL-6 (*P* < 0.0001).

The effects of *ex vivo* and *in vivo* LPS challenge on plasma metabolic parameters, redox balance indicators and inflammation were then measured and listed in Table 6. For the *ex vivo* model, only glucose concentrations were different between conditions with and without LPS (*P* < 0.0001). More specifically, LPS treatment induced a decrease in the medium glucose concentration. *In vivo*, 6 hours after LPS injection, metabolic changes were observed with an increase in the medium glucose concentration (*P* < 0.0001), a decrease in triglyceride concentration (*P* < 0.0001) and a tendancy for uric acid concentration to decrease (*P* = 0.0827). It also affected the redox balance by decreasing the GSH/GSSG ratio (*P* = 0.0130) and GPx activity (*P* = 0.0013) involved in the antioxidant defense.

**Table 6:** Effects of LPS on metabolic parameters, redox balance and inflammation indicators in blood cells incubated for 6 h with LPS (*ex vivo*) or from blood sampled 6 h after LPS subcutaneous injection (*in vivo*).

**Variable**	***Ex vivo***			***In vivo***		
	**Medium**	**LPS**	***p-value***	**T0**	**T6**	***p-value***
**Metabolic parameters**						
Uric acid (mg/L)	21.17 ± 0.93	20.56 ± 0.89	0.6392	50.83 ± 3.90	40.82 ± 3.85	0.0827
Glucose (mg/L)	2,101.5 ± 27.23	1,909.8 ± 16.26	** <0.0001**	2,108.6 ± 26.03	2,458.8 ± 44.60	** <0.0001**
Triglyceride (mg/mL)	403.5 ± 21.08	414.9 ± 23.23	0.7186	158.5 ± 13.23	75.98 ± 7.80	** <0.0001**
**Redox balance**						
TAS (mmol/L)	0.26 ± 0.02	0.28 ± 0.03	0.4196	0.92 ± 0.03	0.88 ± 0.06	0.3909
GSH/GSSG ratio	10.80 ± 0.50	9.76 ± 0.33	0.1106	13.58 ± 0.95	10.41 ± 0.51	**0.0130**
GPx (U/L)	8,739.8 ± 95.14	8,893.1 ± 116.5	0.3202	13,821.6 ± 374.3	12,134.1 ± 243.8	**0.0013**
SOD (U/mL)	14.62 ± 1.05	15.37 ± 1.15	0.6347	26.63 ± 1.05	25.36 ± 1.16	0.3354
TBARS (mmol/mL)	1.33 ± 0.03	1.30 ± 0.03	0.4857	1.29 ± 0.05	1.23 ± 0.04	0.2973
**Inflammation**						
Haptoglobin-like activity (mg/mL)	ND	ND	ND	1.01 ± 0.07	0.85 ± 0.07	0.1167

*Mean values ± SEM (n = 12). Data were analyzed by ANOVA or with non-parametric Mann-Whitney test. Bold values indicate statistically significant differences*.

### Biological Assessment of MEL Extract Supplementation in the *ex vivo* Model of Inflammation and Oxidative Stress

**Figure 3:**
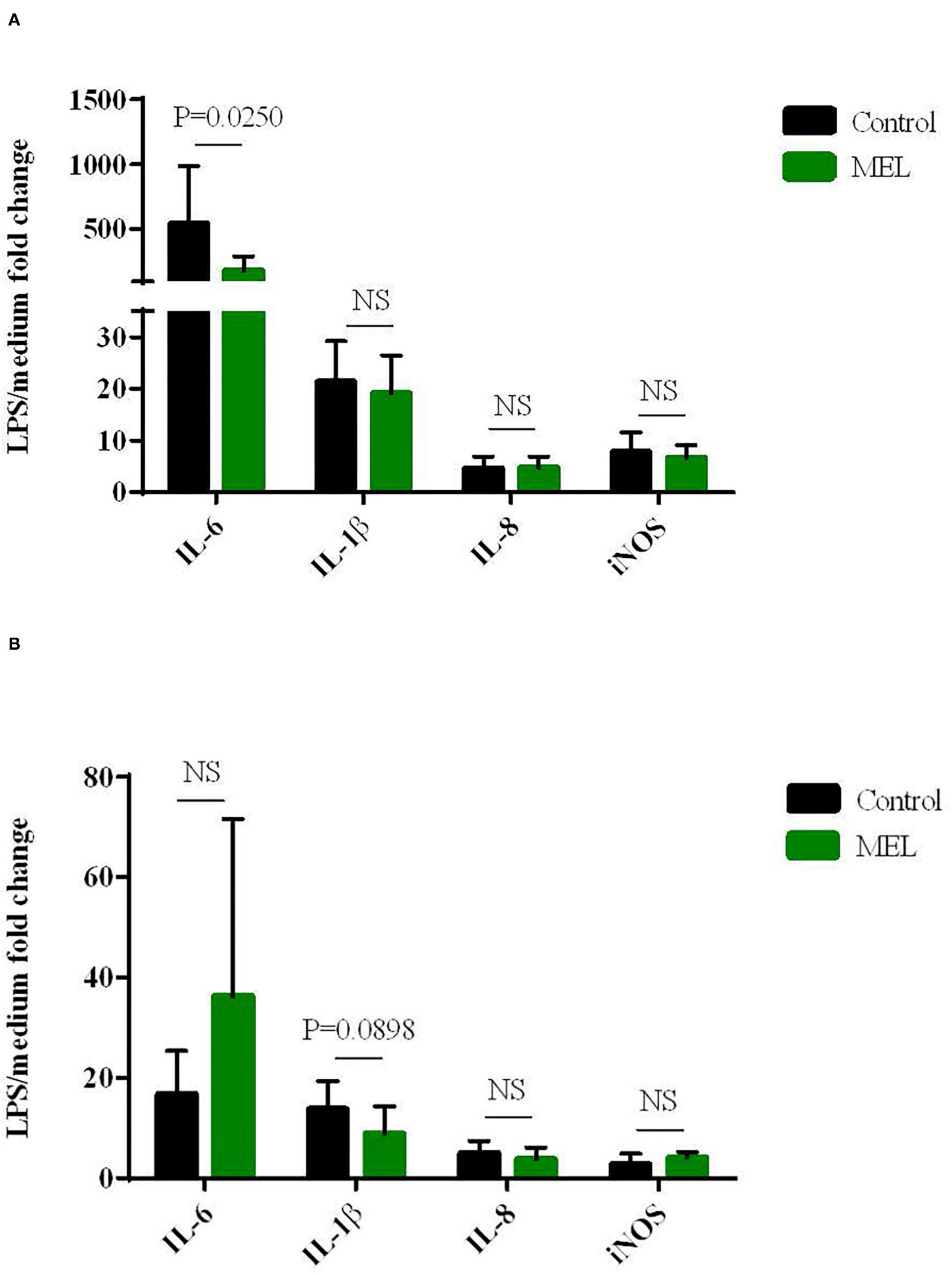
Effects of MEL extract supplementation on the expression of cytokines and iNOS mRNAs in chicken blood cells maintened for 6 hours in culture (*ex vivo*) with LPS (10 μg/mL) at D14 **(A)** and D30 **(B)**. mRNA expression was normalized to GAPDH, HPRT and RPS8 reference genes. Data represent mean ± SEM (*n* = 12). Data were analyzed by ANOVA or with non-parametric Mann-Whitney test. NS = *P* > 0.01.

The effects of MEL extract supplementation in chicken diet on blood cell gene expression *ex vivo* to LPS are shown in Figure 3. At D14, MEL extract supplementation of chicken decreased the gene expression of the pro-inflammatory cytokine IL-6 in blood cells in response to LPS compared to the control blood cells from non-supplemented chicken (*P* = 0.025), but not for IL-8, IL-1β and iNOS (Figure 3A). At D30, MEL supplementation tended only to decrease IL-1β expression (*P* = 0.0898) but without affecting the expression of other genes (Figure 3B).

The direct effects of MEL extract supplementation on plasma metabolic parameters, redox balance and inflammation markers were analyzed at D34. Plasma SOD activity (*P* = 0.0039), uric acid concentration (*P* = 0.0342) decreased and haptoglobin-like activity as a tendancy (*P* = 0.0705) at D34 in chickens eating the feed supplemented with MEL compared to control chickens fed with the basal diet only (Table 7). The GSH/GSSG ratio was significantly higher in chickens with the MEL-supplemented feed (*P* = 0.0087). No significant difference was observed between the two conditions for GPx activity, TAS, TBARS content, glucose or triglyceride concentrations.

**Table 7:** Effects of MEL extract supplementation on physiological parameters of chicken blood at D34.

**Variable**	**Control**	**MEL 1%**	***p-value***
**Metabolic parameters**			
Uric acid (mg/L)	53.20 ± 3.27	44.33 ± 1.98	**0.0342**
Glucose (mg/L)	2,444.6 ± 44.53	2,439.2 ± 17.78	0.9474
Triglyceride (mg/mL)	239.6 ± 13.31	221.8 ± 16.50	0.4071
**Redox balance**			
TAS (mmol/L)	1.07 ± 0.05	0.96 ± 0.04	0.1095
GSH/GSSG ratio	9.24 ± 0.16	9.96 ± 0.19	**0.0087**
GPx (U/L)	13,465.2 ± 241.8	13,322.2 ± 396.9	0.7666
SOD (U/mL)	13.25 ± 2.04	7.78 ± 0.34	**0.0039**
TBARS (mmol/mL)	1.54 ± 0.12	1.34 ± 0.16	0.3299
**Inflammation**			
Haptoglobin-like activity (mg/mL)	0.79 ± 0.06	0.64 ± 0.05	0.0705

*Mean values ± SEM (n = 12). Data were analyzed by ANOVA or with non-parametric Mann-Whitney test. Bold values indicate statistically significant differences*.

### Biological Effects in Chickens Fed With MEL Extract After a Negative Postnatal Experience

No difference between groups was observed on the chick quality evaluated at hatching with the Tona criteria. Moreover, MEL extract supplementation in the feed had no impact on the chick behavior evaluated by the EBENE grid (data not shown).

As shown in Table 8, MEL extract supplementation significantly increased body weight (*P* = 0.0049) and average daily gain (*P* = 0.0022) during the growth period. Despite similar feed intake between the two groups throughout the rearing period, the feed conversion ratio was reduced during the growth period (*P* = 0.0010) showing a better feed efficacy during this period.

**Table 8:** Effects of MEL extract supplementation on zootechnical performance of chickens reared under suboptimal starting conditions.

**Variable**	**Control**	**MEL 1%**	***p-value***
**Body weight (g)**			
1 day of age	38 ± 0.42	38 ± 0.30	0.9556
11 days of age	292 ± 1.58	289 ± 1.45	0.2125
21 days of age	764 ± 3.94	780 ± 4.06	**0.0049**
31 days of age	1,834 ± 9.92	1,823 ± 8.99	0.4454
**Daily weight gain (g/day)**			
1-11 days of age	25.58 ± 0.29	25.21 ± 0.17	0.2878
12-21 days of age	47.28 ± 0.44	49.16 ± 0.42	**0.0022**
22-31 days of age	106.9 ± 1.06	104.3 ± 0.97	0.0737
**Feed intake (g/day)**			
1-11 days of age	28.93 ± 0.36	28.81 ± 0.20	0.7648
12-21 days of age	75.65 ± 0.32	75.59 ± 0.35	0.8972
22-31 days of age	155.2 ± 1.49	153.1 ± 1.01	0.2520
**Feed conversion ratio**			
1-11 days of age	1.13 ± 0.003	1.14 ± 0.005	0.0563
12-21 days of age	1.60 ± 0.012	1.54 ± 0.008	**0.0010**
22-31 days of age	1.44 ± 0.008	1.45 ± 0.008	0.2876

*Mean values ± SEM (n = 280). The means obtained for body weight and daily weight gain were obtained using individual data while the means obtained for feed intake and feed conversion ratio were obtained per pen. Data were analyzed by ANOVA or with non-parametric Mann-Whitney test. Bold values indicate statistically significant differences*.

**Figure 4:**
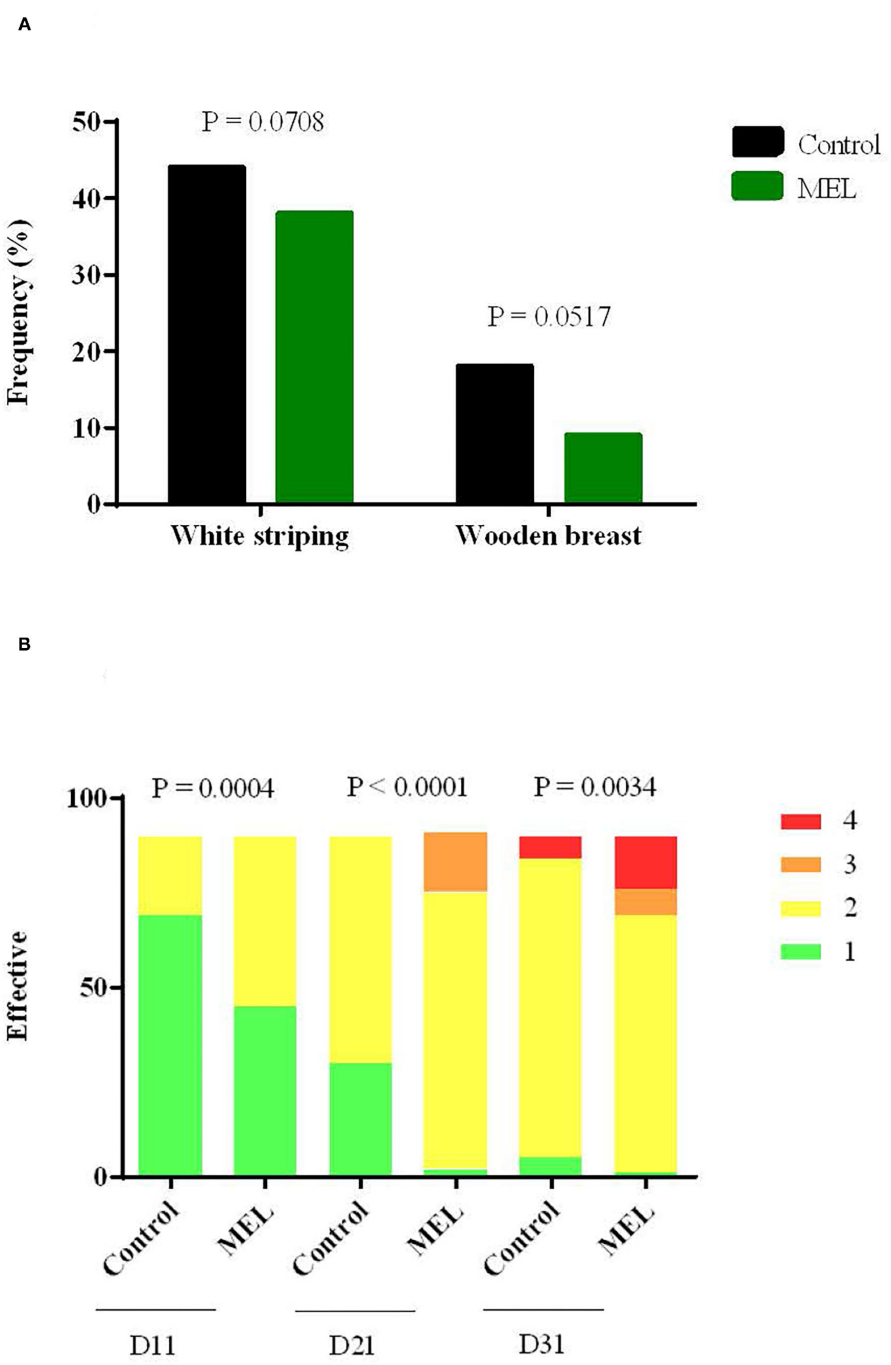
Effects of MEL extract supplementation on muscle defect frequency such as white striping and wooden breast at slaughter age **(A)** and on the severity of lesions for pododermatitis **(B)** at 11, 21, 31 days of age. Data represent the number of animals for each of the muscular defects (*n* = 100) and scores attributed to pododermatitis (*n* = 90), between control and MEL groups (Chi-square test).

Considering health status, MEL extract supplementation had notable effects on occurrence of muscle defects and pododermatitis. The percentage of breast filets with white striping and wooden breast defects tended to be lower in the MEL-supplemented group (Figure 4A). For pododermatitis, the severity of lesions increased with age and significantly increased in chicken supplemented with MEL compared to control chicken at D11, D21 and D31 (Figure 4B). The heterophil/lymphocyte ratio did not differ (*P* = 0.3047) between the two groups (data not shown). The mortality rate was 2% (30/1440 birds) and was not different between groups.

Plasma metabolic parameters and redox balance and inflammation markers analyzed at D30 showed that blood antioxidant status was modified in chicken supplemented with MEL. TAS increased (*P* = 0.0492) while uric acid, glucose, triglyceride concentrations and haptoglobin-like activity did not differ between the two groups (Table 9).

**Table 9:** Effects of MEL extract supplementation on physiological parameters of blood from chickens reared under suboptimal starting conditions at D30.

**Variable**	**Control**	**MEL 1%**	***p-value***
**Metabolic parameters**			
Uric acid (mg/L)	31.86 ± 2.07	33.17 ± 1.96	0.6512
Glucose (mg/L)	2471.6 ± 25.48	2483.7 ± 43.51	0.9713
Triglyceride (mg/mL)	264.2 ± 24.71	244.0 ± 15.32	0.4984
**Antioxidant status**			
TAS (mmol/L)	1.20 ± 0.03	1.36 ± 0.06	**0.0472**
SOD (U/mL)	28.10 ± 3.53	20.38 ± 0.39	0.1222
**Inflammation**			
Haptoglobin-like activity (mg/mL)	2.12 ± 0.10	1.98 ± 0.06	0.2325

*Mean values ± SEM (n = 18). Data were analyzed by ANOVA or with non-parametric Mann-Whitney test. Bold values indicate statistically significant differences*.

## Discussion

The objective of this study was to develop a methodology to assess the ability of an herbal extract to strengthen the innate defenses of poultry. Herbal extracts are mainly used as feed additives to improve performance and to contribute to reducing antimicrobial drugs in poultry. In this study, the approach focused on improving characterization of the ability of an herbal extract to support the functioning of the chicken immune system, especially regarding inflammation and oxidative stress. This was applied to MEL extract, whose biological activities including antioxidant and anti-inflammatory properties have been reported (13–17). MEL is a plant present all over the world and easy to grow and adapt to different environmental conditions (17). Nevertheless, few studies have demonstrated and documented the capacity of MEL extract to improve the defense system of chickens and its impact on their health, welfare and performance (20, 21). The different steps reported in this study range from the quality of MEL extract, its possible cytotoxicity on chicken cell lines and its potential to stimulate immune functions *in vitro*, the capacity to reveal its antioxidative and anti-inflammatory properties in an original *ex vivo* model of inflammation to the assessment of its effects on welfare, health and performance indicators in chickens reared in suboptimal conditions.

Phytochemical investigations on the MEL extracts used in this study revealed the presence of bioactive substances including phenolic compounds such as dihydroxycinnamid acid derivatives and a major proportion of free sugars and maltodextrin. As the highest level among the phenolic compounds, RA and its quantification by HPLC methods is considered to be an indicator of the MEL extract quality (16). In the MEL extracts used in this study, the proportion of RA was quantified between 1 and 2 % (10–20 mg/g extract) as would be expected (16) and remained stable after 9 months of storage. This proportion is below 5% RA declared by the suppliers probably because the method commonly used is not HPLC but spectrophotometry detecting ortho diphenol compounds as RA and others. The use of reference chromatographic fingerprints combined with quantitation of pharmacologically active compounds of the herbal extract recommended by pharmacopeias are useful when these molecules are present in the range of 0.1 to 10 mg per gram of extract. It may not be sufficient when these methods of quantification are applied to feed supplemented with herbal extracts. RA used as a tracer of MEL extract was evaluated in the different feed used in this study and detected as expected in starter and grower but not in finisher feed (Design 2). The supplement rate of MEL extract in feed (1%) was still in the range of HPLC-DAD and RF methods used but the difference between the theorical and the analyzed RA content in the feed (60 to 80%) could be related to the interference of many constituents of the feed mash with the analytical procedure of RA. According to Kerkora et al. (35), a proposed mechanism for these interferences is an oxidation step of the phenolic groups of RA to quinones followed by linkage with N or S groups of the amino acids present in the proteins or as free amino acids.

However it is remarkable that the RA concentration in feed was still stable 3 months after MEL extract supplementation in starter and grower feed (experiment 2). The development of analytical methods to reach the ppm range is required with purification and concentration steps in order to lower these potential interferences. The choice of tracers and methods to detect them should also progress in the future by using new technologies to characterize better the quality of herbal extracts and their traceability during the process of feed production (36).

To evaluate the potential of herbal extracts to support innate immunity, the phytochemical composition gives information on potential pharmacological activities. The major compounds of MEL exhibiting an antioxidant activity are phenolic compounds, which possess the ability to scavenge free radicals involved in lipid peroxidation, and to improve plasma levels of catalase, superoxide dismutase and glutathione peroxidase (14). The quantification of antioxidant activity is well described (14, 37) but information about its biological antioxidant activity *in vivo* is lacking. For pro- or anti-inflammatory properties, it is necessary to use cell systems (38, 39) or to experiment *in vivo* to characterize them. In chickens, models of oxidative stress and inflammation are directly performed *in vivo* (40–43). To reduce animal experimentation, replacement alternative methodologies are encouraged [the 3Rs principle; (44, 45)]. *In vitro* methods using chicken hepatocyte and macrophage cell lines were used in this study to assess the potential cytotoxicity and immunostimulant properties of the MEL extract. The MEL extract did not induce cytotoxic effects on the HD11 (macrophage) or LMH (hepatocyte) cell lines at 6, 24 or 48 h of incubation. However, a significant decrease in metabolic activity was observed in macrophages after 48 h of incubation for the highest concentration (100 μg/mL). In a study conducted by Moacă et al. (18), MEL leaf extracts were selectively more cytotoxic at 24 h of incubation against tumor cells (MDA-MB-231) than against healthy cells (100 μg/mL vs. 500-1000 μg/mL) and no cytotoxicity was observed on primary culture of porcine liver cells (PLP2) incubated with MEL extracts at 400 μg/mL (46). The use of macrophage cell lines to evaluate the capacity of herbal extracts to regulate redox balance and inflammation is relevant and the transcription factor NF-κB works as a link between oxidative-induced damage and inflammation (47). Interestingly, MEL extract (for 100 and 10 μg/mL) stimulated NF-κB activity and NO production by avian macrophages in a manner relatively similar to that induced by LPS. Hence, MEL extract seems to have immunostimuling properties in chicken macrophages.

The next step was to develop a method to highlight MEL antioxidant and anti-inflammatory properties on primary chicken cells. LPS is commonly used to induce cell inflammation *in vivo* and *in vitro*. In the present study, we developed an *ex vivo* method to trigger inflammation and oxidative stress in chicken blood cells incubated with LPS and compared it to a subcutaneous injection of LPS in chicken (25). *Ex vivo* LPS challenge of blood cells promoted the upregulation of pro-inflammatory cytokines, mainly IL-6 and to a lower extent IL-1β, IL-8, and iNOS enzyme involved in NO synthesis. These results are in agreement with those previously reported for macrophage cell lines (39, 48). *In vivo*, the subcutaneous injection of LPS induced a predominant upregulation of IL-8 and to a lesser extent it up regulated IL-1β and IL-6 but not iNOS. These results are complementary to those obtained in chicken spleen cells (25). The amplification of the gene expression was remarkably higher in *ex vivo* than in *in vivo* methods. This could be explained by the fact that the cells mobilized after LPS challenge *in vivo*, such as heterophils and macrophages, were no longer present in the blood when the sample was taken (6 h after the injection) and had probably migrated to the site of injection or adhered to activated post capillary venules (49). Regarding these two approaches, the *ex vivo* method on chicken blood cells enabled the expression of inflammation and oxidative stress biomarkers and had the advantage of allowing the use of blood from chicken without any cell purification and avoiding LPS injection in animals. The inflammatory response requires a strong mobilization of energy reserves in organisms which was observed by an increase in blood glucose concentration and a decrease in triglyceride concentration after LPS injection in chicken but not in the *ex vivo* method. Moreover, it was accompanied by a decrease in the GSH/GSSG ratio, evidence of oxidative stress, and in GPx activity, an antioxidative enzyme that scavenges various peroxides (50). Altogether, both *ex vivo* and *in vivo* methods provide indicators of inflammation and oxidative stress that can be used to assess the biological activities of herbal extracts, with a preference for the *ex vivo* method.

To be more relevant to the physiological interaction of MEL extract with the whole chicken, the *ex vivo* method was conducted on blood cells collected from chickens fed with or without MEL extract. This method showed that blood cells from chickens fed with MEL extract expressed a lower inflammatory response than those from control chickens to LPS. The lower IL-6 expression observed at D14 and a trend for lower IL-1β expression at D30 from blood cells are in agreement with the anti-inflammatory response observed with macrophages stimulated by LPS and incubated *in vitro* with *Angelica gigas* compound (39). The originality in this method is to reveal the biological activity of MEL extract *in vivo* via the *ex vivo* use of chicken blood cells. These results were complemented by the direct detection in plasma of a lower concentration of the acute phase haptoglobin-like protein and more specifically a lower uric acid concentration, a lower SOD activity and a higher GSH/GSSG ratio in favor of a better antioxidative status in chickens fed with MEL extract.

The last step was to assess the effects of the plant extract in a real situation observed in livestock. Previous studies reproducing a delayed placement of chicks as could occur on farms showed negative effects on performance, with immediate and long-term modifications of the redox balance in the blood (4, 51). Based on these studies, feed supplementation with MEL was tested in chickens reared in experimental delayed placement conditions. The beneficial effect of MEL extract consumption on the redox balance was confirmed by an increase in the total antioxidative status (TAS) and a lower SOD activity in the chicken blood at the end of the rearing period (D30). Interestingly, the performance of these chickens was improved during the grower phase with also a trend for a lower occurrence of muscle defects. These effects of MEL extract supplementation on chicken performance have been described previously (20, 21) and are strengthened by our study. The beneficial antioxidative activity of MEL was also reported on the quality of meat by limited lipid oxidation (20). However, despite no negative effects being observed in chicken welfare, the severity of pododermatitis lesions, although moderate, increased with age and in chickens supplemented with MEL extract compared to control chickens at D11, D21 and D31. The effects of MEL extracts on digestive enzymes (52) and traditional uses of MEL leaves for their digestive, carminative, antispasmodic and diuretic properties (14) could impact the intestinal transit of chickens and fecal consistency including urine. The choice of 1% MEL extract supplementation in feed was based on a few experimental studies (20, 21). A “dose effect” of MEL extract incorporation in feed would be relevant for its use in real farm breeding situations. This study in chickens reared in suboptimal conditions with MEL supplementation is essential to assess a multicriteria analysis including all parameters of interest such as health, welfare and performance and not only to focus on the expected properties of MEL extract. This is also a step required before validating the beneficial effects of feed supplementation with herbal extracts on farms.

## Conclusions

Different and complementary methods are required to reveal the beneficial use of bioactive herbal extracts in poultry feed. This study proposes a common methodological pipeline which could be adapted to the plant extract chosen and the biological effects expected. The complementarity of the methods and protocols ensures reliability and robustness for the observed effects. The *in vitro* and *ex vivo* methods should be favored before experimenting on chickens but they will not replace the assessment of plant extacts in the animal. These methods and tools could be combined according to the needs of professionals working in poultry production systems and staff in charge of animal health, welfare and feeding.

## Data Availability Statement

The original contributions presented in the study are included in the article/supplementary material, further inquiries can be directed to the corresponding author/s.

## Ethics Statement

The animal study was reviewed and approved by the local Ethics Committee N°019 (Comité d'Ethique en Expérimentation Animale Val de Loire, Tours, France) and the Animal Experimentation Ethics Committee N°073 (Aquitaine Poissons Oiseaux, France).

## Author Contributions

LG, AT, and RG supervised the study. LG, AT, RG, CB-C, MP, and FS designed and developed the experiments. AP, LG, AT, RG, DB, and CB-C wrote the first draft of the manuscript. All authors contributed to the technical work, the data analyses and to manuscript revision and they read and approved the submitted version.

## Funding

This study was supported by the CASDAR (project 2016–2020 MEXAVI) funded by the French Ministry of Agriculture. The authors would also like to thank the CIPC (Comité Interprofessionnel du Poulet de Chair) for supporting the project.

## Conflict of Interest

The authors declare that the research was conducted in the absence of any commercial or financial relationships that could be construed as a potential conflict of interest.

## Publisher's Note

All claims expressed in this article are solely those of the authors and do not necessarily represent those of their affiliated organizations, or those of the publisher, the editors and the reviewers. Any product that may be evaluated in this article, or claim that may be made by its manufacturer, is not guaranteed or endorsed by the publisher.
